# The comparative effectiveness of fourth‐line drugs in resistant hypertension: An application in electronic health record data

**DOI:** 10.1002/pds.4808

**Published:** 2019-07-16

**Authors:** Sarah‐Jo Sinnott, Liam Smeeth, Elizabeth Williamson, Pablo Perel, Dorothea Nitsch, Laurie A. Tomlinson, Ian J. Douglas

**Affiliations:** ^1^ Department of Non‐communicable Disease Epidemiology London School of Hygiene and Tropical Medicine London UK; ^2^ Department of Medical Statistics London School of Hygiene and Tropical Medicine London UK

**Keywords:** anti‐hypertensive drugs, comparative effectiveness, high blood pressure, hypertension, pharmacoepidemiology, resistant hypertension

## Abstract

**Purpose:**

To examine the utility of electronic health records from a routine care setting in assessing comparative effectiveness of fourth‐line anti‐hypertensive drugs to treat resistant hypertension.

**Methods:**

We conducted a cohort study using the Clinical Practice Research Datalink: a repository of electronic health records from UK primary care. We identified patients newly prescribed fourth‐line anti‐hypertensive drugs (aldosterone antagonist , beta‐blocker, or alpha‐blocker). Using propensity score–adjusted Cox proportional hazards models, we compared the incidence of the primary outcome (composite of all‐cause mortality, stroke, and myocardial infarction) between patients on different fourth‐line drugs. AA was the reference drug in all comparisons. Secondary outcomes were individual components of the primary outcome, blood pressure changes, and heart failure. We used a negative control outcome, Herpes Zoster, to detect unmeasured confounding.

**Results:**

Overall, 8639 patients were included. In propensity score–adjusted analyses, the hazard ratio for the primary outcome was 0.81 (95% CI, 0.55‐1.19) for beta‐blockers and 0.68 (95% CI, 0.46‐0.96) for alpha‐blockers versus AA. Findings for individual cardiovascular outcomes trended in a more plausible direction, albeit imprecise. A trend for a protective effect for Herpes Zoster across both comparisons was seen.

**Conclusions:**

A higher rate of all‐cause death in the AA group was likely due to unmeasured confounding in our analysis of the composite primary outcome, supported by our negative outcome analysis. Results for cardiovascular outcomes were plausible, but imprecise due to small cohort sizes and a low number of observed outcomes.

1

**KEY POINTS**

- We compared the effectiveness of fourth‐line alpha‐blockers and beta‐blockers to aldosterone antagonists in resistant hypertension. Aldosterone antagonists (AA) were the reference because they were found to be the most effective fourth‐line drug at lowering blood pressure in a recent trial.
- Effectiveness was measured by a composite primary outcome: all‐cause death, myocardial infarction, and stroke. Secondary outcomes included the individual components of the primary outcome, heart failure, and changes in blood pressure. We used a negative control outcome to help identify if confounding/bias was present.
- We found that those exposed to alpha‐blockers and beta‐blockers were at a decreased, albeit imprecise, risk of the primary outcome in comparison to those exposed to aldosterone antagonists. A higher rate of all‐cause death in the AA group was likely due to unmeasured confounding in our analysis of the composite primary outcome, supported by our negative outcome analysis.
- Results for cardiovascular outcomes and blood pressure changes were plausible, indicating less confounding for specific outcomes.

## 2 INTRODUCTION

Hypertension, or high blood pressure (BP), is a leading risk factor for cardiovascular and cerebrovascular deaths.^1^ These deaths constitute more than 30% of all deaths globally, and with hypertension being highly prevalent, have been declared a global public health crisis.^2^, ^3^ Resistant hypertension (RH) is defined as BP that remains ≥140/90mmHg despite being treated with maximum, or best tolerated doses, of three or more anti‐hypertensive drugs, one of which should be a diuretic.^4^, ^5^, ^6^ Almost 7% of the treated hypertensive population in the United Kingdom has RH, representing approximately 800 000 people.^7^ Those with RH have worse health outcomes than those with “standard” hypertension, which double the risk of cardiovascular events.^8^ Thus, the prevention and treatment of RH is of great importance in reducing the burden of cardiovascular disease and mortality.^1^, ^9^

RH has traditionally been an area of unmet treatment need.^10^ However, PATHWAY‐2, a recent clinical trial, of 285 patients with RH has provided evidence that spironolactone, an aldosterone antagonist (AA) with diuretic activity, is better at reducing BP in comparison to a beta‐blocker,an alpha‐blocker, 11 The trial, although badly needed, was somewhat limited in that it looked at reductions in BP as opposed to “hard” clinical outcomes of major interest such as myocardial infarction, stroke, and death. Furthermore, patients in the trial were followed for 12 weeks, which is a short amount of time given that the complications of high BP develop over longer time periods. Such limitations are inherent in many randomised trials where financial costs, logistics, and ethical considerations often mean larger scale trials with longer follow up are not feasible.

Patients, care providers, and regulators are increasingly seeking detailed evidence of medication effects in routine care settings, but optimal, valid methods for conducting this kind of research are currently uncertain.^12^ Electronic health record (EHR) data offer an opportunity to determine whether the comparative effectiveness of fourth‐line anti‐hypertensive drugs can be investigated in a routine care setting.^13^ Data for large heterogeneous populations allow capture of rare outcomes such as myocardial infarction, stroke, and death over longer periods of time than that can be typically used in randomised controlled trials. However, different anti‐hypertensive drugs can be used preferentially depending on a patient's adverse drug event profile, their comorbidities, and physician preference.^14^, ^15^ Whether EHR data allow accurate capture of this confounding by indication remains to be examined.

Thus, we used EHR data to study how BP changes following initiation of different fourth‐line anti‐hypertensive drugs and to assess whether we can reliably use routine care data to inform on long‐term clinical outcomes in this context.

## 3 METHODS

### 3.1 Study design and data

We conducted a retrospective cohort study, using the Clinical Practice Research Database (CPRD‐GOLD), a nationally representative repository of de‐identified EHRs from primary care in the United Kingdom. CPRD‐GOLD holds data on demographic information, health‐related behaviours, test results including BP readings, diagnoses, and prescriptions for more than 11 million people in more than 670 practices across the United Kingdom since 1987.^16^ It is one of the largest databases of longitudinal medical records from primary care globally and has been extensively validated.^17^ Data quality are monitored by CPRD internal processes.

### 3.2 Cohort

Patients were eligible for cohort entry from the latest of practice up‐to‐standard date, patients current registration date plus 1 year, patients' 18th birthday, or study start 2 February 1998. We identified patients who initiated a fourth‐line anti‐hypertensive, AA, beta‐blocker, or alpha‐blocker between 1998 and 2016. To mirror guideline‐defined RH,^14^ we required that the patient's base regimen comprised an angiotensin converting enzyme‐inhibitor/angiotensin receptor blocker (ACE‐I/ARB), calcium channel blocker (CCB), and a thiazide diuretic. To resemble the PATHWAY‐2 clinical trial^11^ and to minimise confounding by indication, we applied the following exclusion criteria: BP <140/90mmHg serum potassium >5.5mmol/L, pulse rate <55 or > 120 beats per minute, estimated glomerular filtration rate (eGFR) <45mL/min/1.73m^2^, or a diagnosis of heart failure. To ensure that patients were continuing concurrent treatment with all four drugs as opposed to switching, we required repeat prescriptions of all four drugs within 6 months of initiating the fourth‐line drug. The date on which continued use of four drugs was confirmed was referred to as the index date.

We attempted to exclude patients who displayed poor medication adherence behaviour, because this has been noted as a main cause of many cases of apparent RH.^18^, ^19^ In the absence of dispensing records, which are typically used to measure adherence at the population level, we instead used prescribing records to estimate a proxy for drug adherence. We measured this proxy for each patients' drug regimen in the 1‐year period prior to initiating the fourth‐line drug. Using prescription dates and computed days' supply prescribed, we calculated proxy adherence as the number of days covered by the drug divided by the number of days in the observation period. We accounted for leftover days' supply from previous prescriptions by adding to the next supply. We calculated average adherence across all three drugs and then categorised as adherent or not based on an 80% threshold.^20^ If patients did not meet our definition for proxy adherence, they were excluded.

### 3.3 Outcomes

The primary outcome was a composite of first myocardial infarction, stroke, and all‐cause mortality. This three‐component composite outcome is frequently used in trials of cardiovascular outcomes and is statistically helpful in instances where low event rates might occur for a single outcome.^21^, ^22^ Secondary outcomes included change in systolic BP, heart failure, end‐stage renal failure, myocardial infarction, stroke, and all‐causemortality. All‐cause mortality was included because it is assumed to be an “ideal” endpoint given that it can be determined without ascertainment bias and that preventing mortality is the ultimate goal of many drug treatments. Adverse outcomes were hyperkalaemia defined as serum potassium ≥6 mmol/Land gynecomastia. We used incidence of Herpes Zoster as a negative control outcome to explore unmeasured confounding between fourth‐lineanti‐hypertensive groups (see the Supporting Information for codes used to identify all outcomes).^23^

### 3.4 Covariates

We used data on the following covariates: age, sex and lifestyle factors; smoking, alcohol use, and body mass index. The closest records to fourth‐lineanti‐hypertensive initiation date were used for determining lifestyle factors using existing algorithms.^24^ Other medication use included prior use of statins, anti‐platelet agents, proton pump inhibitors, insulin, and loop diuretics. We also captured medication usage from multiple British National Formulary chapters in the year prior to initiation to indicate polypharmacy. We categorised as usage of drugs from 0 to 4 chapters, 5 to 8 chapters, and ≥9 chapters. We accounted for the following comorbidities: diabetes, prior myocardial infarction, prior stroke, arrhythmia, peripheral vascular disease, cancer, depression, and chronic obstructive pulmonary disease. To capture health service use, we constructed a variable indicating how often a patient used primary care services in the year prior to initiation. This was categorised as 0 to 9 consultations, 10 to 19 consultations, 20 to 29 consultations, 30 to 39 consultations, and ≥40 consultations. We calculated baseline eGFR (45‐60mL/min/1.73m^2^ or >60mL/min/1.73m^2^) using the most recent creatinine value from CPRD data 1 year prior to fourth‐linedrug initiation and the Chronic Kidney Disease Epidemiology Collaboration (CKD‐EPI) equation.^25^ Serum potassium at baseline was categorised as <5mmol/Lor 5‐5.5mmol/L. We included morbidities indicative of a secondary cause of hypertension: phaeochromocytoma, sleep apnoea, aldosteronism, Cushing's syndrome, and renal causes. We also included drugs that are known to increase BP in the year prior to initiation date: non‐steroidalanti‐inflammatorydrugs, tacrolimus/ciclosporin, erythropoietin, high dose steroids (equivalent to 20‐mgprednisolone daily) for at least 2 weeks, and the oral contraceptive pill. Lastly, we included information on symptoms and testing that could suggest a presence of heart failure, shortness of breath, peripheral oedema, and evidence of echocardiograph. We described data for ethnicity but did not include in analytical models due to more than 50% missingness.^26^

### 3.5 Statistics

We analysed each drug comparison separately: beta‐blockers versus AA and alpha‐blockers versus AA. There were approximately 20 to 25% missing data for baseline categories of eGFR and serum potassium and approximately 5% missing data for lifestyle variables: smoking, alcohol consumption, and body mass index. To maximise sample size, we imputed missing data under the missing at random assumption.^27^ In the imputation model, we included all explanatory variables listed above, including the outcome variable and the Nelson‐Aalenestimate of the cumulative hazard to the survival time for each individual outcome assessment.^27^, ^28^ We conducted diagnostics using the midiagplots function in Stata.^29^ Within each of the 10 imputed datasets and using all the covariates listed above, we calculated a propensity score, wherein AA was the reference group for drug comparison (Appendix A). We then used this propensity score in an adjusted Cox Proportional Hazards model to estimate the hazard ratio and 95% confidence interval for each outcome, and then combined treatment effects across each imputed dataset to get one overall estimate.^30^ For changes in systolic BP, we used cubic spline mixed models with a random intercept for each patient. Such models allow for correlations within patients for BP results and also accommodate the unbalanced nature of BP readings in the data.^31^ There was a median of 17 (IQR 9‐29 BP measurements available during follow‐up for each patient. Patients without a BP measurement during follow‐upwere dropped from the BP analysis (n=133).

In all analyses, follow‐up started on the index date, ie, the date on which use of four concurrent anti‐hypertensive drugs was confirmed. Follow‐upcontinued until the patient experienced an outcome, death, withdrew from the general practice, last data collection date for each practice, end of study (February 2016) or 3‐yearpost index date, whichever occurred first. All main analyses were intention to treat.

### 3.6 Sensitivity and subgroup analyses

We conducted subgroup analyses, whereby the main analysis was stratified by age, gender, diabetes, CKD, and baseline systolic BP. We also conducted analyses where we applied (1) further PATHWAY‐2 exclusion criteria and (2) less stringent exclusion criteria removing criteria relating to BP, serum potassium, pulse rate, eGFR, and diagnosed heart failure.^11^ We also conducted stratified analyses according to arrhythmia at baseline given that some of the drugs used to meet the definition of RH could also be used to treat arrhythmias. We carried out a sensitivity analysis, whereby follow‐up started from date of initiation (ie, the date of fourth drug initiation) as opposed to from index date (date on which continued use of four drugs was confirmed). Our rationale was that this analysis would capture adverse events and outcomes directly after initiation in patients who may not have had a repeat prescription of the fourth drug helping us to understand, to some degree, the number of events our main analysis may have missed. An extension of this analysis considered BP changes in patients who had evidence of repeat prescriptions of fourth‐linedrugs, but with follow‐upbeginning on date of initiation. This analysis aimed to assess whether BP changes in this observational cohort are similar to those found in PATHWAY‐2.^11^ We also conducted sensitivity analyses in which we censored follow‐upwhen the patient discontinued their fourth‐lineanti‐hypertensivedrug or switched to a different fourth‐linedrug. Finally, we conducted a complete‐caseanalysis including only patients who did not require imputation of covariates.

## 4 RESULTS

**Figure 1:**
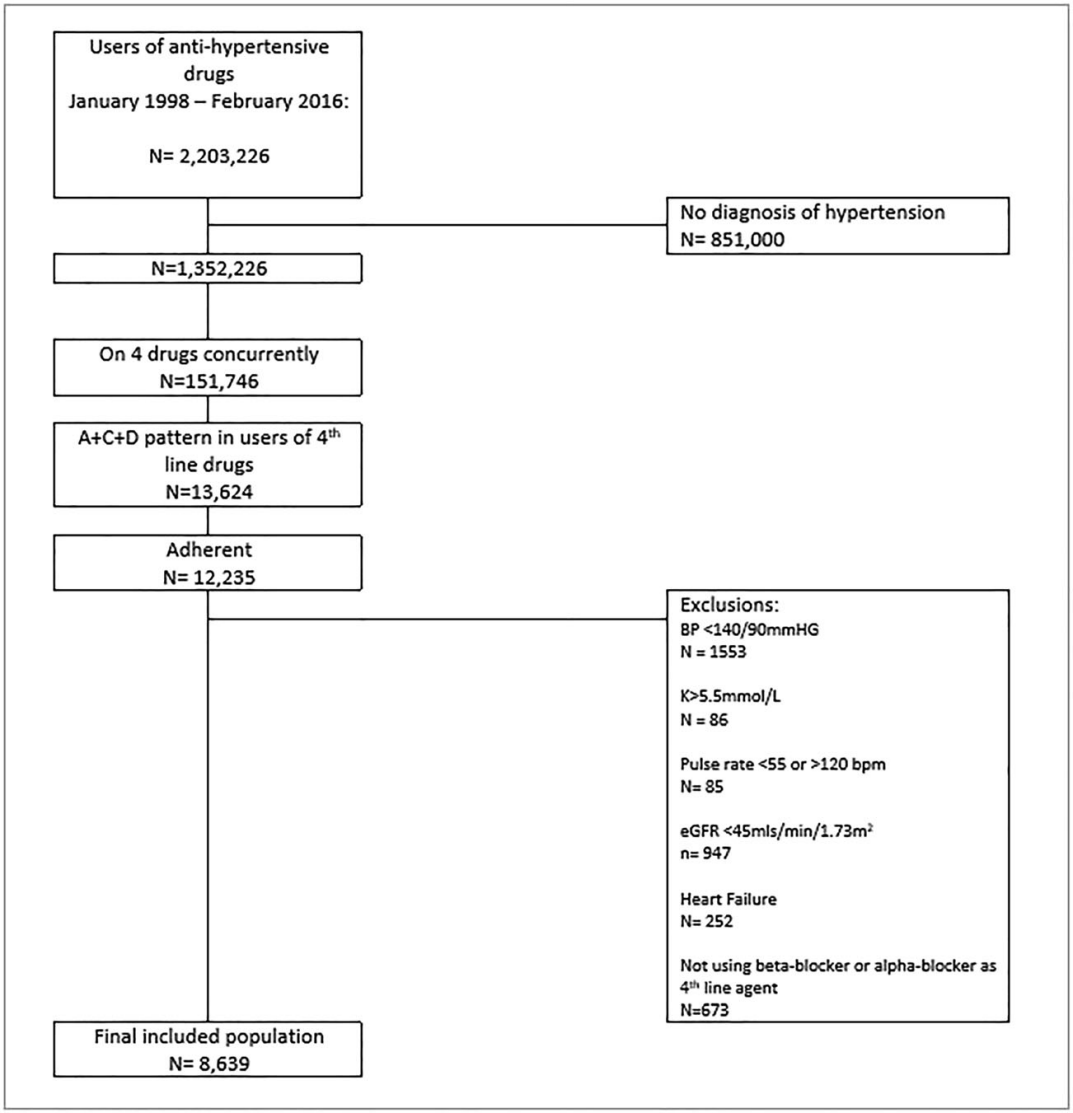
A flowchart demonstrating study inclusion and exclusion criteria

From more than 2 million users of anti‐hypertensive drugs, we identified 8639 people who were treated with an ACE‐I/ARBplus a CCB plus a diuretic prior to addition of fourth‐lineanti‐hypertensive drug (Figure 1). The mean age of included patients was 64.9 years (SD 11.2), and the population was 43.4% female. Diabetes was most prevalent in patients initiating alpha‐blockers (Table 1). Those initiating an AA as a fourth‐linedrug had the highest prevalence of tests/symptomsindicating heart failure, eg, evidence of an echocardiograph, shortness of breath, and peripheral oedema. This group also had the highest prevalence of non‐cardiovascularcomorbidities. Systolic and diastolic BP at initiation of fourth‐lineanti‐hypertensivewere highest in patients initiating alpha‐blockers (Table 1). A table comparing common baseline characteristics for PATHWAY‐2 and this observational cohort is provided in Appendix B in the Supporting Information.

**Table 1:** Baseline characteristics of patients initiating fourth‐line anti‐hypertensive drugs

	Alpha‐blockers, %	Aldosterone Antagonist n, %	Beta‐blocker n, %
No of Patients	5420	350	2869
Females	2244 (41.4)	182 (52.0)	1324 (46.1)
Age, Years			
<50	580 (10.7)	33 ( 9.4)	279 ( 9.7)
50‐59	1184 (21.8)	67 (19.1)	549 (19.1)
60‐64	823 (15.2)	45 (12.9)	379 (13.2)
65‐69	954 (17.6)	58 (16.6)	464 (16.2)
70‐74	780 (14.4)	58 (16.6)	474 (16.5)
75‐79	603 (11.1)	44 (12.6)	391 (13.6)
80+	496 ( 9.2)	45 (12.9)	333 (11.6)
Ethnicity			
White	2177 (40.2)	142 (40.6)	1172 (40.9)
South Asian	51 (0.9)	na	35 (1.2)
Black	90 (1.7)	6 (1.7)	34 (1.2)
Other/mixed	30 (0.6)	na	13 (0.5)
Missing	3072 (56.7)	199 (56.9)	1615 (56.3)
Smoking			
Non‐smoking	1913 (35.3)	136 (38.9)	1145 (39.9)
Current smoker	870 (16.1)	32 (9.1)	417 (14.5)
Ex‐smoker	2452 (45.2)	161 (46)	1232 (42.9)
Missing	185 (3.4)	21 (6)	75 (2.6)
Alcohol			
Non‐drinking	612 (11.3)	38 (10.9)	331 (11.5)
Current drinker	3917 (72.3)	240 (68.6)	2094 (73)
Ex drinker	565 (10.4)	42 (12)	268 (9.3)
Missing	326 (6)	30 (8.6)	176 (6.1)
Body mass index (kg/m^2^)			
Underweight <18.5	19 (0.4)	na	10 (0.4)
Healthy_weight 18.5‐24.9	729 (13.5)	40 (11.4)	466 (16.2)
Overweight 25‐29.9	1764 (32.6)	93 (26.6)	977 (34.1)
Obesity ≥30	2629 (48.5)	190 (54.3)	1259 (43.9)
Missing	279 (5.2)	24 (6.9)	157(5.5)
Comorbidities			
Myocardial Infarction	141 (2.6)	17 (4.9)	131 (4.6)
Stroke	377 (7.0)	29 ( 8.3)	224 (7.8)
Peripheral vascular disease	382 (7.0)	23 (6.6)	147 (5.1)
Diabetes	1939 (35.8)	115 (32.9)	743 (25.9)
Depression	444 (8.2)	42 (12.0)	203 (7.1)
COPD	309 (5.7)	28 (8.0)	90 (3.1)
Cancer	527 (9.7)	46 (13.1)	309 (10.8)
Secondary causes of hypertension^a^	173 (3.2)	23 (6.6)	75 (2.6)
Indicators of possible heart failure			
Echocardiograph	528 ( 9.7)	73 (20.9)	332 (11.6)
Shortness of breath	845 (15.6)	111 (31.7)	355 (12.4)
Peripheral oedema	389 ( 7.2)	35 (10.0)	210 ( 7.3)
eGFR (mL/min)			
≥60	3488 (64.4)	220 (62.9)	1736 (60.5)
45‐59	992 (18.3)	70 (20.0)	523 (18.2)
Missing	940 (17.3)	60 (17.1)	610 (21.3)
Drugs			
Antiplatelet	2420 (44.6)	166 (47.4)	1241 (43.3)
Statins	3055 (56.4)	204 (58.3)	1479 (51.6)
Proton pump inhibitors	1793 (33.1)	158 (45.1)	983 (34.3)
Insulin	439 ( 8.1)	27 ( 7.7)	148 ( 5.2)
Loop diuretic	705 (13.0)	85 (24.3)	334 (11.6)
BP increasing drugs^b^	234 ( 4.3)	18 ( 5.1)	113 ( 3.9)
Number of unique consultations		
0‐9	1947 (35.9)	82 (23.4)	1056 (36.8)
10‐19	2335 (43.1)	144 (41.1)	1187 (41.4)
20‐29	757 (14)	88 (25.1)	407 (14.2)
30‐39	211 (3.9)	16 (4.6)	120 (4.2)
≥40	170 (3.1)	20 (5.7)	99 (3.5)
Number of unique BNF chapters			
0‐4	3043 (56.1)	157 (44.9)	1669 (58.2)
5‐8	2108 (38.9)	158 (45.1)	1085 (37.8)
≥9	269 (5.0)	35 (10.0)	115 (4.0)
Physiological parameters mean (SD)			
Potassium	4.27 (0.46)	4.15 (0.45)	4.28 (0.45)
Missing n, %	1144 (21.1)	76 (21.7)	715 (24.8)
Systolic BP	163.1 (15.9)	161.8 (16.6)	161.2 (16.9)
Missing n, %	45 (0.8)	11 (3.1)	54 (1.9)
Diastolic BP	86.4 (12.4)	84.8 (12.5)	85.6 (12.6)
Missing n, %	45 (0.8)	11 (3.1)	54 (1.9)
Pulse rate	78.9 (13.1)	79.1 (13.8)	84.1 (14.6)
Missing n, %	4694 (86.3)	278 (79.4)	2392 (83.1)

*Note*. “na” refers to cell sizes too small to report in accordance with our data agreements.

Abbreviations: BNF, British National Formulary; BP, blood pressure; COPD, chronic obstructive pulmonary disease; SD, standard deviation.

Phaeochromocytoma, sleep apnoea, aldosteronism, Cushing's syndrome, and renal causes measured using all available data.

Non‐steroidal anti‐inflammatory drugs, tacrolimus/ciclosporin, erythropoietin, high dose steroids (equivalent to 20mg prednisolone daily for at least 2 weeks), and the oral contraceptive pill in the 365‐day period prior to initiation.

### 4.1 Primary outcome analyses

In separate analyses comparing beta‐blockersand alpha‐blockersto AA, a protective effect for the primary outcome (composite of first myocardial infarction, stroke, and all‐causemortality) was observed across both comparisons, although the 95% confidence intervals (95% CI) approached the null. After adjusting for the propensity score, there was no change in the direction of the hazard ratios and the 95% CI continued to cross or approach the null (Table 2). Within 3 years of index date 115/2869 (4.0%) users of beta‐blockers had died, 241/5420 (4.4%) users of alpha‐blockers had died, and 24/350 (6.9%) users of AA had died.

**Table 2:** Crude and adjusted hazard ratios for the primary outcome

	N	Outcomes	Crude HR (95% CI)	Adjusted HR (95% CI)
Beta‐blockers vs aldosterone antagonists	2827	204	0.69 (0.47‐0.99)	0.81 (0.55‐1.19)
Alpha‐blockers vs aldosterone antagonists	5215	334	0.63 (0.44‐0.91)	0.68 (0.46‐0.96)

Crude: age‐ and gender‐adjusted only.

*Note*. Adjusted: propensity score adjusted.

Abbreviation: HR, hazard ratio.

### 4.2 Secondary Outcome analyses

#### 4.2.1 BP changes

**Figure 2:**
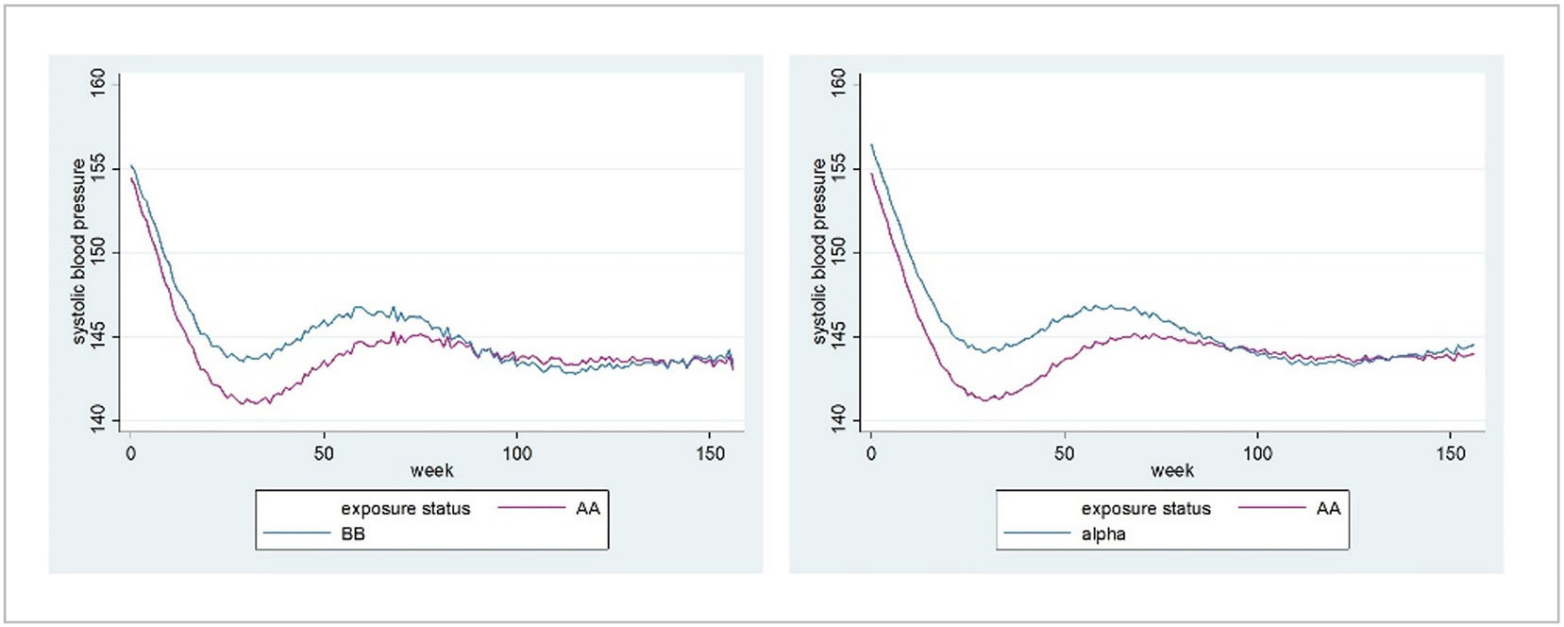
Three‐year blood pressure changes from index date for (A) beta‐blockers vs aldosterone antagonists and (B) alpha‐blockers vs aldosterone antagonists [Colour figure can be viewed at wileyonlinelibrary.com]

At 12 weeks, systolic BP was approximately 2mmHg higher in the beta‐blockerand alpha‐blockergroups in comparison to AA (Figure 2 and Table 3). However, by 2‐yearfollow‐up, there was a negligible difference in systolic BP. From initiation date (as opposed to index date), there were differences of approximately 3mmHg in the beta‐blockerand alpha‐blockergroups in comparison to AA, but by 2 years, there was negligible difference (Appendix C).

**Table 3:** Systolic blood pressure from index date

	% with BP Readings	Beta‐blockers Systolic BP mmHg (95% CI)	% with BP Readings	Aldosterone Antagonists Systolic BP mmHg (95% CI)
At baseline	100	155.2 (154.6‐155.8)	100	154.4 (152.6‐156.2)
At 12 week	76.0	147.8 (147.2‐148.4)	72.0	146.1 (144.4‐147.7)
At 1 year	96.1	145.9 (145.2‐146.5)	94.0	143.5 (141.7‐145.3)
At 2 years	98.0	143.5 (142.9‐144.1)	96.3	143.9 (142‐145.7)
At 3 years	98.3	143.4 (142.5‐144.3)	96.9	143.1 (140.4‐145.7)
		Alpha‐blockers Systolic BP mmHg (95% CI)		Aldosterone Antagonists Systolic BP mmHg (95% CI)
At baseline	100	156.4 (156‐156.9)	100	154.7 (153‐156.5)
At 12 weeks	80.1	148.6 (148.2‐149)	72.0	146.2 (144.6‐147.8)
At 1 year	97.4	146.2 (145.8‐146.6)	94.0	143.7 (142‐145.5)
At 2 years	98.6	143.8 (143.3‐144.2)	96.3	144 (142.3‐145.8)
At 3 years	98.7	144.5 (143.9‐145.1)	96.9	144 (141.4‐146.6)

*Note*. Weekly and yearly time points refer to time passed since index date. Data are from propensity score–adjusted cubic spline mixed models.

Abbreviations: BP, blood pressure; CI, confidence interval.

#### 4.2.2 Secondary clinical outcomes

A trend towards increased stroke and heart failure for those initiating beta‐blockersand alpha‐blockers in comparison to AA was observed, although the 95% CI encompassed the null effect (Table 4). In contrast, a trend towards decreased death for users of beta‐blockersand alpha‐blockerswas observed, but again, the 95% CI encompassed the null effect. The number of outcomes observed for end stage renal disease was low (Appendix D), and thus, we did not formally analyse.

**Table 4:** Hazard ratios and 95% CI for secondary outcomes and negative outcome in each fourth‐line anti‐hypertensive comparison

	Beta‐blockers vs Aldosterone Antagonists				Alpha‐blockers vs Aldosterone Antagonists	
	N	Outcome	HR (95% CI)	n	Outcome	HR (95% CI)
Death	3217	142	0.68 (0.41‐1.11)	5768	271	0.65 (0.42‐1.01)
Stroke	2960	58	2.50 (0.57‐10.91)	5353	78	2.64 (0.63‐11.05)
MI	3059	46	0.95 (0.37‐2.46)	5607	86	1.05 (0.45‐2.48)
Heart failure	3192	67	2.68 (0.82‐8.81)	5759	98	2.35 (0.71‐7.80)
Negative outcome: Herpes Zoster	2983	55	0.67 (0.28‐1.58)	5400	94	0.78 (0.34‐1.78)

*Note*. Analyses are propensity score adjusted.

Abbreviation: HR, hazard ratio; MI, myocardial Infarction.

### 4.3 Adverse outcomes

There was protective effect for hyperkalaemia when beta‐blockersand alpha‐blockerswere compared with AA (Appendix E). The number of outcomes observed for gynecomastia was low (Appendix D), and we did not formally analyse.

### 4.4 Negative outcome

Although imprecise, there was a trend towards a protective effect when beta‐blockersand alpha‐blockerswere compared with AA for Herpes Zoster (Table 4).

### 4.5 Subgroup and sensitivity analyses

The 95% CI for all subgroup analyses overlapped with the 95% CI for the main effect; however, there was a trend for those who were <60years and those who had diabetes to have increased hazard for the primary outcome than those without, and this was generally consistent across both drug comparisons (Appendix F). There was no strong evidence to suggest a difference in hazard for the primary outcome when stratified by arrhythmia at time of initiation (Appendix G). In a sensitivity analysis, we censored patients when they either discontinued their fourth‐linedrug or started another fourth‐linedrug. The confidence limits overlapped with those in the main analysis (Appendix H). We also examined the hazard for the primary outcome and adverse outcomes from initiation date (as opposed to index date). For the primary outcome, the protective effect observed for the primary outcome was exaggerated for those initiating beta‐blockersand alpha‐blockersin comparison to the main analysis (Appendix I). For adverse outcomes, the results were similar to those reported from index date (Appendix E). The results of the complete‐caseanalysis were similar to the main analysis (Appendix J).

## 5 DISCUSSION

In this cohort study of 8639 patients with RH, we found inconclusive results for the association between beta‐blockersand alpha‐blockerscompared with AA and the occurrence of the combined outcome of myocardial infarction, stroke, and all‐causemortality. For secondary outcomes (heart failure, myocardial infarction, stroke, and all‐causemortality), the magnitude and direction of the hazard ratios for some cardiovascular endpoints appeared plausible, although imprecise due to low numbers of outcomes. The results for all‐causemortality suggest the presence of uncontrolled confounding, a finding that was supported by the negative control outcome analysis.

We found that patients prescribed AA as a fourth‐lineantihypertensive in RH had systolic BP values approximately 2mmHg lower than those in patients prescribed beta‐blockersand alpha‐blockersat 12‐weekpost index date, and 3mmHg lower post initiation date. In the PATHWAY‐2 clinical trial, an average difference of 4mmHg (clinic BP) for the same comparisons was found, averaged across 6‐and 12‐weekfollow‐upvisits.^11^ Over the duration of follow‐upin our study, the differences in systolic BP between the comparison drugs diminished to negligible for the beta‐blockerand alpha‐blockercomparisons.

Other observational studies have compared different fourth‐lineanti‐hypertensivedrugs and found reductions in systolic BP of on average 12mmHg, favouring AA.^32^ These previous observational studies were carried out in hospitals; thus, the identification and inclusion processes were not similar to the algorithms we used. Additionally, investigators in both randomized controlled trials and cohort studies using primary data collection have control over the frequency and method of BP measurement, which is not possible in EHR data. As seen from Table 3, between 70 and 80% of patients in our study had BP measurements within 12 weeks of index date.

Based on the findings of PATHWAY‐2, and our findings for BP reductions, it could be reasonably expected that cardiovascular outcomes occur at a lower rate in those exposed to AA. 11, 33 Our results for the primary outcome do not reflect this expectation. Rather than detract from the utility of data from routine care settings to carry out comparative effectiveness analyses, it is important to explore why our study did not produce the expected results.

We pragmatically used a composite primary outcome to achieve an adequate number of events for a powered analysis. However, including all‐causemortality in the primary outcome lead to a confounded association. Our negative control analysis, whilst having low statistical power, implied a lower risk of herpes zoster with beta‐blockersand alpha‐blockerswhen compared with AA, when no association should be expected, reaffirming our suspicion of residual confounding. In other words, users of AA were, at baseline, different in terms of their morbidity profile and at greater risk of death than users of the other medication groups. This problem is in theory redeemable if information on factors associated with both the exposure and closeness to death (ie, confounders) can be measured and adjusted for, eg, undiagnosed heart failure or frailty. We attempted to adjust for undiagnosed heart failure in our analyses by using proxy variables, breathlessness, peripheral oedema, and history of echocardiography. These covariates were imbalanced between the medication groups, with higher prevalence among AA users. Unfortunately, these symptoms/testsare likely to be a limited proxy for heart failure and may not be coded completely, thus limiting their potential in confounding adjustment. We believe that frailty was imbalanced between the medication groups as indicated by the higher prevalence of several comorbidities, medication utilisation, and health service utilisation covariates in the AA group compared with the other medication groups. Information on key indicators of frailty is not readily available in EHR data, eg, grip strength, and leads to incomplete capture of this important confounding mechanism between the exposure and all‐causedeath.^34^

In contrast, the direction of our results for some individual cardiovascular outcomes appeared plausible, albeit imprecise. Imprecision is likely a direct result of small cohort sizes and a low number of outcomes. Our cohort sizes may have been conservatively small; in the absence of a formal diagnosis code, we applied a strict definition for RH based on medication usage, diagnosis codes for hypertension, and an estimate of proxy adherence, along with multiple exclusion criteria to remove patients who may have been using a similar medication regimen for the treatment of heart failure. Our method of measuring adherence likely underestimated true adherence,^35^, ^36^ however was the most pragmatic option available in the absence of linked dispensing data.

An additional limitation to this study is that those with RH are not a straightforward population to isolate from EHR data. Indeed, even in clinical settings, identification of those with true RH is challenging.^18^, ^19^ Therefore, it is possible that some of the included patients were not “true” RH patients; some may have had the medication patterns we required and may have had coded hypertension but may also have been using some anti‐hypertensivedrugs for indications other than hypertension. We carried out multiple sensitivity analyses to test our identification process and to mimic the PATHWAY‐2 population as closely as possible; however, these yielded results similar to the main analysis.

Lastly, we did not include data on ethnicity in our propensity score models due to missingness.^26^ However, the CPRD population is representative of the UK population in terms of ethnicity. Furthermore, in a sensitivity analysis restricted to people of white ethnicity, we found similar results to the main analysis.

Despite the above limitations, some strengths do exist. This is the first study, randomised or observational, to examine the comparative effectiveness of fourth‐linedrugs in RH with regard to the incidence of clinical outcomes. We conducted a range of additional analyses, which help to understand the limitations of our approach and help to signpost future efforts that could improve upon our study.

## 6 CONCLUSION

We used EHR data to investigate the comparative effectiveness of different fourth‐lineanti‐hypertensivedrugs used to treat RH in routine care using an observational design with propensity score adjustment. The findings of a recent clinical trial, PATHWAY‐2, imply that outcomes should occur at a lower rate in those exposed to AA in comparison to other fourth‐lineanti‐hypertensivedrugs.^11^ We found inconclusive results for the primary outcome. We suspect that this occurred due to unmeasured confounding.

Conversely, the direction and magnitude of results for some secondary cardiovascular outcomes did not appear to be confounded to the same extent and are somewhat more plausible, albeit with wide confidence intervals.

Despite our findings, addressing this research question in routine health care data is not without future potential. Next efforts using alternative data sources, data linkage for better capture of comorbidities diagnosed or managed in secondary care, and further methodological development such as more complete capture of data on characteristics such as frailty, which may help overcome confounding.

## ETHICS STATEMENT

The study protocol was approved by the London School of Hygiene and Tropical Medicine ethics committee (no. 13988) and the independent scientific advisory committee for Medicines and Healthcare products Regulatory Agency (No 17_247Mn).

## CONFLICT OF INTEREST

The authors declare no conflict of interest.

## FUNDING

S. J. S. was funded by a Sir Henry Wellcome Fellowship (107340/Z/15/Z) from Wellcome Trust. I. J. D. reports grants from GlaxoSmithKline outside the submitted work and has shareholdings in GSK. Laurie Tomlinson was funded by a Wellcome Trust intermediate clinical fellowship (101143/Z/13/Z). L. S. reports grants from GSK, during the conduct of the study; grants from Wellcome (098504/Z/12/Z); grants from MRC; grants from NIHR; grants from BHF; grants from Diabetes UK, outside the submitted work; and a Trustee of the British Heart Foundation. D. N. reports grants from Informatica Systems and grants from GSK, outside the submitted work.

## Supporting information

Appendix A: Graphs of Propensity Score created for primary outcome analyses
Appendix B: Baseline Characteristics of PATHWAY‐2 and this observational cohort
Appendix C: Blood pressure changes from initiation date
Appendix D: Numbers of events
Appendix E: Adverse outcomes
Appendix F: Subgroup Analyses
Appendix G: Stratified on arrhythmia at baseline
Appendix H: Discontinuation or addition/switchof a 4^th^ line agent
Appendix I: Primary outcome since initiation date
Appendix J: Complete Case Analysis
Appendix K: Analysis restricted to patients with coded white ethnicity
