# Supplementary material for: The comparative effectiveness of fourth‐line drugs in resistant hypertension: An application in electronic health record data
Source: Pharmacoepidemiol Drug Saf. 2019 Jul 16;28(9):1267–77. doi: 10.1002/pds.4808 (PMC6771826; doi:10.1002/pds.4808)
Supplement: Supplementary file 1 — Appendix A: Graphs of Propensity Score created for primary outcome analyses Appendix B: Baseline Characteristics of PATHWAY‐2 and this observational cohort Appendix C: Blood pressure changes from initiation date Appendix D: Numbers of events Appendix E: Adverse outcomes Appendix F: Subgroup Analyses Appendix G: Stratified on arrhythmia at baseline Appendix H: Discontinuation or addition/switchof a 4th line agent Appendix I: Primary outcome since initiation date Appendix J: Complete Case Analysis Appendix K: Analysis restricted to patients with coded white ethnicity [file PDS-28-1267-s001.docx]

## Supplementary Material

### Appendix 1: Graphs of Propensity Score created for primary outcome analyses

*
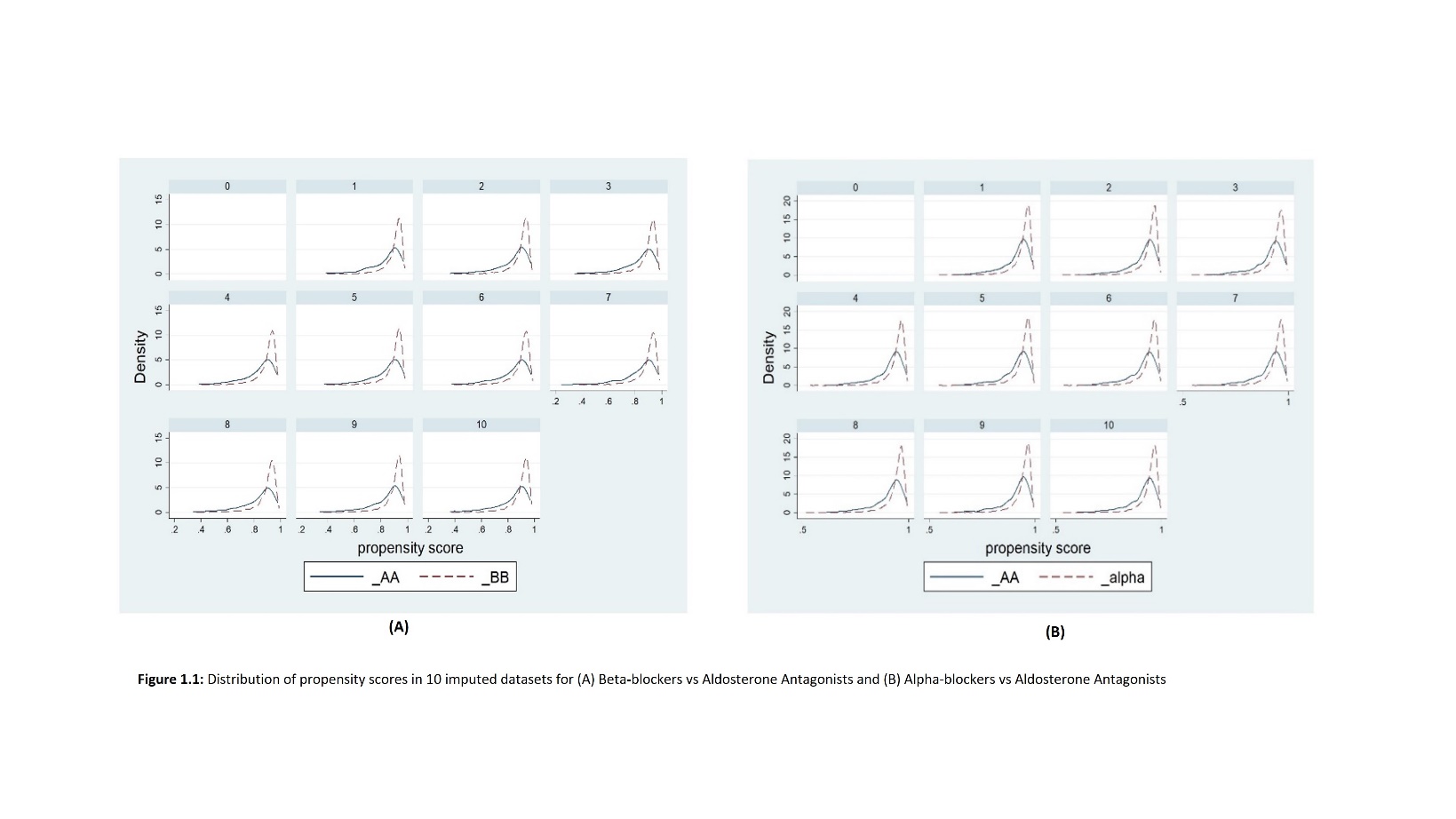
*

### Appendix 2: Baseline Characteristics of PATHWAY-2 and this observational cohort

| **Table 2.1** Baseline Characteristics in PATHWAY-2 and this observational cohort | | |
| --- | --- | --- |
|  | PATHWAY-2 | Observational cohort |
|  | n= 335 | n= 8639 |
| **Age - mean(sd)** | 61.4 (9.6) | 64.9 (11.2) |
| **Female - n (%)** | 105 (31.0) | 3750 (43.4) |
| **Smoking - n (%)** | 26 (7.8) | 1319 (15.3) |
| **Missing - n (%)** | na | 306 (3.3) |
| **Diabetes - n (%)** | 46 (14.0) | 2,797 (32.4) |
| **Mean eGFR mls/min - mean (sd)** | 91.1 (26.8) | 74.6 (16.6) |
| **Missing - n (%)** | na | 1610 (18.6) |
| **Baseline Clinic Systolic BP - mean (sd)** | 157.0 (14.3) | 162.4 (16.3) |
| **Missing - n (%)** | Na | 110 (1.3) |
| **Baseline Clinic Diastolic BP - mean (sd)** | 90.0 (1.5) | 86.1(12.5) |
| **Missing - n (%)** | Na | 110 (1.3) |

### Appendix 3: Blood pressure changes from initiation date

| **Table 3.1:** Systolic blood pressure from initiation date | | |
| --- | --- | --- |
|  | **Beta-blockers mmHg (95% CI)** | **Aldosterone**  **Antagonists mmHg (95% CI)** |
| At baseline | 156.3 (155.7 - 157) | 156 (154.1 - 157.8) |
| At 12 weeks | 149.1 (148.5 - 149.6) | 146.4 (144.7 - 148) |
| At 1 year | 146.1 (145.5 - 146.7) | 143.8 (141.9 - 145.6) |
| At 2 years | 143.4 (142.8 - 144) | 143.3 (141.4 - 145.1) |
| At 3 years | 143.2 (142.4 - 144.1) | 143.5 (140.8 - 146.1) |
|  |  |  |
|  | **Alpha-blockers mmHg (95% CI)** | **Aldosterone Antagonists mmHg (95% CI)** |
| At baseline | 157.8 (157.4 - 158.3) | 156.2 (154.4 - 157.9) |
| At 12 weeks | 149.6 (149.2 - 150) | 146.5 (145 - 148.1) |
| At 1 year | 146.5 (146.1 - 146.9) | 144 (142.3 - 145.8) |
| At 2 years | 143.8 (143.4 - 144.2) | 143.6 (141.8 - 145.3) |
| At 3 years | 144.3 (143.7 - 144.9) | 143.8 (141.3 - 146.3) |

*Notes: This analysis was carried out in patients who had concurrent use of 4 anti-hypertensive drugs. Follow-up started at initiation date rather than index date for this analysis.*

## Appendix 4: Numbers of events

| **Table 4.1:** Number of events for end stage renal disease and gynecomastia | | |
| --- | --- | --- |
| **End Stage Renal Disease** | **n** | **outcomes** |
| Beta-blockers vs Aldosterone Antagonists | 3215 | 8 |
| Alpha-blockers vs Aldosterone Antagonists | 5763 | 17 |
|  |  |  |
| **Gynecomastia** |  |  |
| Beta-blockers vs Aldosterone Antagonists | 3205 | 6 |
| Alpha-blockers vs Aldosterone Antagonists | 5754 | 12 |

## Appendix 5: Adverse outcomes

| **Table 5.1:** Adverse events from index and initiation dates | | |
| --- | --- | --- |
|  | **Hyperkalaemia since index** | **Hyperkalaemia since initiation** |
| **Beta-blockers** **vs Aldosterone Antagonists** | n= 3204, outcomes = 212 | n= 26410, outcomes = 1925 |
|  | 0.30 (0.22 - 0.42) | 0.28 (0.26 - 0.31) |
| **Alpha-blockers vs Aldosterone Antagonists** | n= 5745, outcomes = 326 | n= 52287, outcomes = 2858 |
|  | 0.26 (0.20 - 0.34) | 0.22 (0.20 -0.24) |

Notes: numbers are larger for the “from initiation” analyses because initiators were people who initiated a 4^th^ line agent, but di

## Appendix 6: Subgroup Analyses


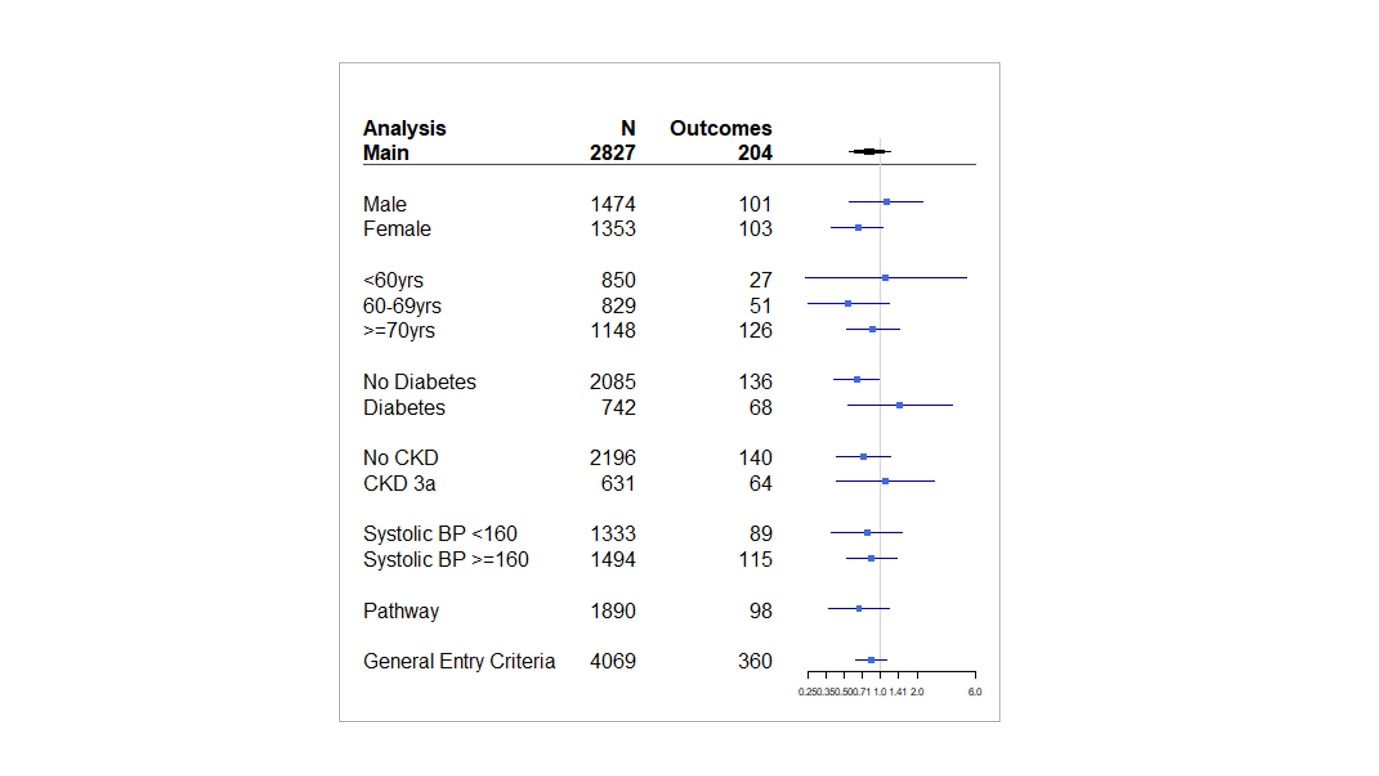


**Figure 6.1:** Subgroup Analyses for Beta-blockers vs Aldosterone Antagonists. Hazard Ratios and 95% CI are presented for the primary outcome (myocardial infarction, stroke and death).

All parameters measured at baseline.

Pathway refers to replicating the Pathway Clinical Trial inclusion criteria as far as possible. In addition to the main exclusions we applied for the main analysis we excluded the following; patients who used a blood pressure raising drug in the year prior to initiation (NSAIDS, immunosuppressants, high dose corticosteroids for duration of 2 weeks, oral contraceptive pill, erythropoietin), patients with a secondary cause of hypertension (phaeochromocytoma, sleep apnoea, aldosteronism, Cushing’s syndrome and renal causes), patients using insulin (as a proxy for type 1 diabetes), patients with a diagnosis code for arrhythmia, patients with a diagnosis code for cancer, and patients aged 80 years at or before treatment initiation.

General entry criteria refers to no requirement for the following exclusions: blood pressure <140/90mmHg; serum potassium >5.5mmol/L; pulse rate <55 or > 120 beats per minute; estimated glomerular filtration rate (eGFR) <45mls/min/1.73m^2^; or a diagnosis of heart failure.

**
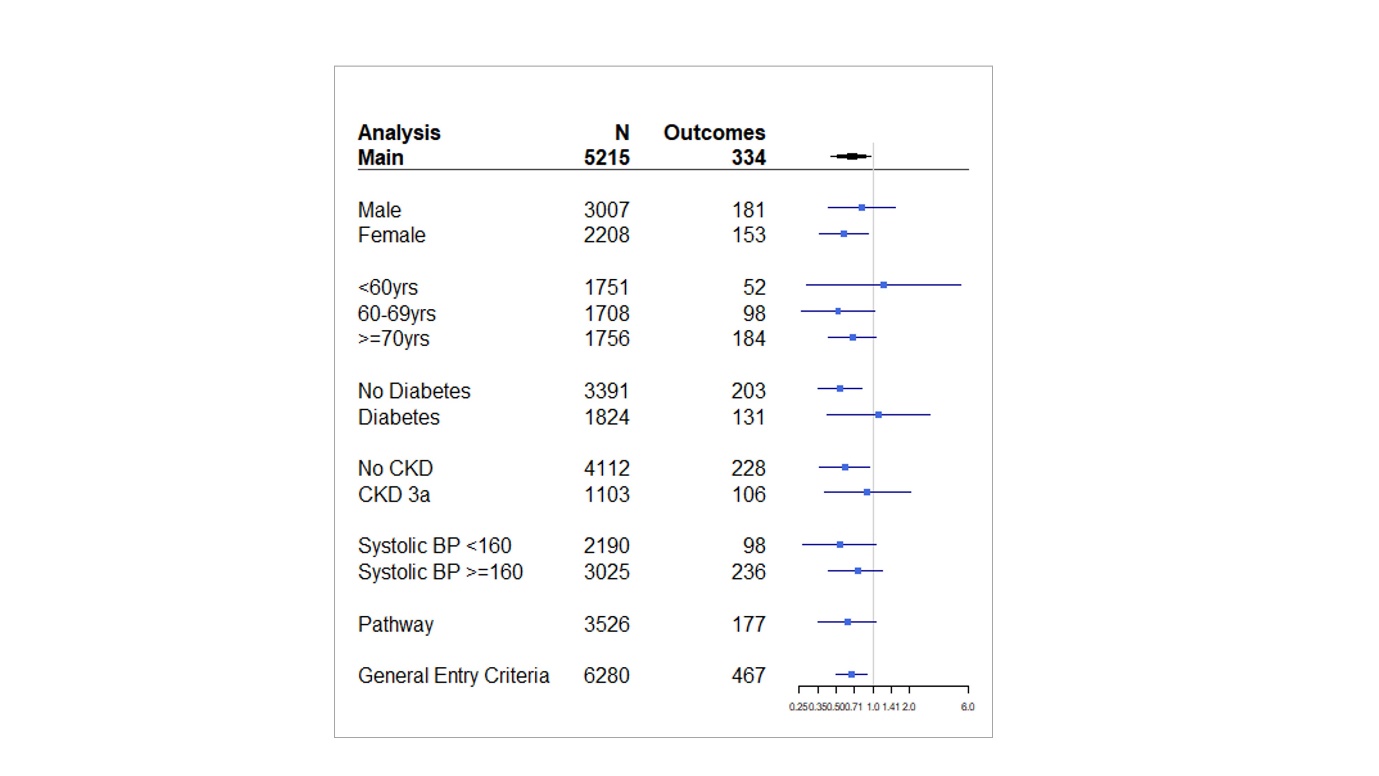
**

**Figure 6.2:** Subgroup Analyses for Alpha-blockers vs Aldosterone Antagonists. Hazard Ratios and 95% CI are presented for the primary outcome (myocardial infarction, stroke and death).

All parameters measured at baseline.

Pathway refers to replicating the Pathway Clinical Trial inclusion criteria as far as possible. In addition to the main exclusions we applied for the main analysis we excluded the following; patients who used a blood pressure raising drug in the year prior to initiation (NSAIDS, immunosuppressants, high dose corticosteroids for duration of 2 weeks, oral contraceptive pill, erythropoietin), patients with a secondary cause of hypertension (phaeochromocytoma, sleep apnoea, aldosteronism, Cushing’s syndrome and renal causes), patients using insulin (as a proxy for type 1 diabetes), patients with a diagnosis code for arrhythmia, patients with a diagnosis code for cancer, and patients aged 80 years at or before treatment initiation.

General entry criteria refers to no requirement for the following exclusions: blood pressure <140/90mmHg; serum potassium >5.5mmol/L; pulse rate <55 or > 120 beats per minute; estimated glomerular filtration rate (eGFR) <45mls/min/1.73m^2^; or a diagnosis of heart failure.

## Appendix 7: Stratified on arrhythmia at baseline

| **Table 7.1:** Hazard Ratios and 95% CI for the primary outcome when stratified by arrhythmia at baseline | | | | | | |
| --- | --- | --- | --- | --- | --- | --- |
|  | **n** | **outcomes** | **Arrhythmia at baseline**  **Hazard Ratio 95% CI** | **n** | **outcomes** | **No arrhythmia at baseline**  **Hazard Ratio 95% CI** |
| **Beta-blockers vs Aldosterone Antagonists** | 181 | 23 | 1.01 (0.27 – 3.79) | 2646 | 181 | 0.76 (0.51 – 1.14) |
| **Alpha-blockers vs Aldosterone Antagonists** | 222 | 31 | 0.79 (0.23 – 2.68) | 4993 | 303 | 0.65 (0.45 – 0.95) |

Notes: Primary outcome is a composite of myocardial infarction, stroke and death. Analyses are fully adjusted with propensity score.

## Appendix 8: Discontinuation or addition/switch of a 4^th^ line agent

| **Table 8.1:** Hazard Ratios and 95% CI for the primary outcome when censored at discontinuation or switching/addition of other exposure/comparator 4^th^ line agents. | | | | | | |
| --- | --- | --- | --- | --- | --- | --- |
|  | **n** | **outcomes** | **Discontinuation**  **Hazard Ratio 95% CI** | **n** | **outcomes** | **Switch/Addition of other exposure/comparator**  **Hazard Ratio 95% CI** |
| **Beta-blockers vs Aldosterone Antagonists** | 2827 | 161 | 0.93 (0.55 – 1.55) | 2044 | 143 | 0.55 (0.34 – 0.90) |
| **Alpha-blockers vs Aldosterone Antagonists** | 5215 | 223 | 0.72 (0.43 – 1.20) | 4864 | 269 | 0.47 (0.29 – 0.75) |

*Notes: Primary outcome is a composite of myocardial infarction, stroke and death. Analyses adjusted for age and sex only.*

### Appendix 9: Primary outcome since initiation date

| Table 9.1: Primary outcome since index date and initiation date | | |
| --- | --- | --- |
|  | **Primary outcome since index** | **Primary outcome since initiation** |
| Beta-Blockers vs Aldosterone Antagonists | n= 2827, outcomes = 204 | n= 22488, outcomes = 2259 |
|  | 0.81 (0.55 - 1.19) | 0.40 (0.37 - 0.44) |
| Alpha-blockers vs Aldosterone Antagonists | n= 5215, outcomes = 334 | n= 46,611, outcomes = 3722 |
|  | 0.68 (0.46 - 0.96) | 0.33 (0.30 - 0.36) |

Notes: numbers are larger for the “from initiation” analyses because initiators were people who initiated a 4^th^ line agent, but did not necessarily continue to be a concurrent user of 4 agents in the patterns we required. Initiation analyses are adjusted for age and sex only

### Appendix 10: Complete Case Analysis

| **Table 10.1:** Crude and Adjusted Hazard Ratios for the Primary Outcome in a Complete-Case analysis | | | | | | | | | |
| --- | --- | --- | --- | --- | --- | --- | --- | --- | --- |
|  | **Crude** | | | | **Adjusted** | | | | |
|  | **n** | **outcomes** | | **HR (95% CI)** | **n** | | **outcomes** | | **HR (95% CI)** |
| Beta-blockers vs Aldosterone antagonists | 2,827 | | 204 | 0.69 (0.47 – 0.99) | 1,943 | 130 | | 0.92 (0.56 – 1.52) | |
| Alpha-blockers vs Aldosterone antagonists | 5,215 | | 334 | 0.63 (0.44 – 0.91) | 3,833 | 222 | | 0.75 (0.47 – 1.20) | |

*Crude: age and gender adjusted only*

*Adjusted: propensity score adjusted*

*Patients only included in this analysis if no missing data for any baseline covariate, disregarding ethnicity.*

### Appendix 11: Analysis restricted to patients with coded white ethnicity

| **Table 10.1:** Crude and Adjusted Hazard Ratios for the Primary Outcome in patients with coded white ethnicity | | | | | | | | | |
| --- | --- | --- | --- | --- | --- | --- | --- | --- | --- |
|  | **Crude** | | | | **Adjusted** | | | | |
|  | **n** | **outcomes** | | **HR (95% CI)** | **n** | | **outcomes** | | **HR (95% CI)** |
| Beta-blockers vs Aldosterone antagonists | 1,154 | | 66 | 0.61 (0.32 – 1.17) | 1,154 | 66 | | 0.75 (0.38 – 1.48) | |
| Alpha-blockers vs Aldosterone antagonists | 2,098 | | 109 | 0.57 (0.32 – 1.02) | 2,098 | 109 | | 0.73 (0.39 – 1.35) | |

*Crude: age and gender adjusted only*

*Adjusted: propensity score adjusted*
